# Breast Cancer Risk Assessment: A Review on Mammography-Based Approaches

**DOI:** 10.3390/jimaging7060098

**Published:** 2021-06-12

**Authors:** João Mendes, Nuno Matela

**Affiliations:** Faculdade de Ciências, Instituto de Biofísica e Engenharia Biomédica, Universidade de Lisboa, 1749-016 Lisboa, Portugal; jpmendes@fc.ul.pt

**Keywords:** breast cancer, risk assessment, machine learning, deep learning, texture, mammography

## Abstract

Breast cancer affects thousands of women across the world, every year. Methods to predict risk of breast cancer, or to stratify women in different risk levels, could help to achieve an early diagnosis, and consequently a reduction of mortality. This paper aims to review articles that extracted texture features from mammograms and used those features along with machine learning algorithms to assess breast cancer risk. Besides that, deep learning methodologies that aimed for the same goal were also reviewed. In this work, first, a brief introduction to breast cancer statistics and screening programs is presented; after that, research done in the field of breast cancer risk assessment are analyzed, in terms of both methodologies used and results obtained. Finally, considerations about the analyzed papers are conducted. The results of this review allow to conclude that both machine and deep learning methodologies provide promising results in the field of risk analysis, either in a stratification in risk groups, or in a prediction of a risk score. Although promising, future endeavors in this field should consider the possibility of the implementation of the methodology in clinical practice.

## 1. Introduction

One in eight women will be diagnosed with breast cancer (BC) in their lifetime, with one in thirty-nine women dying from this disease just in the USA. In the same country, in 2020, approximately 42,170 women were expected to die from BC and it was anticipated that approximately 30% of the cancers detected in women will be BC [1]. Around 95% of cancers are due to genetic mutations that result from environmental or lifestyle factors, where the remaining percentage is related to inherited genes—with BRCA1/BRCA2 genes being responsible for most of cases of BC [2,3].

BC diagnosis occurs either during a common screening program, before symptoms appear, or after women noticing some breast changes. Screening programs are important for an early detection of BC—that is, in a more treatable stage—resulting in a decrease in mortality [1,4].

The criterion that defines if a woman is eligible for screening is, normally, only her age. Different countries have different recommendations on which age is the best to start screening; the USA states that women from age 45 to 54 should have a mammography once a year, while 55+ plus women should have a mammography once every two years. On the other hand, the UK NHS says that only women between 50 to 71 should be screened, and only once every three years [5,6].

Although there are multiple screening programs, they might not serve all women. Some younger women may be at higher risk of developing breast cancer than women in their fifties and, despite that, these women are not eligible for screening. With that in mind, the perfect screening program should not consider age as the only risk factor that determines when to screen women.

The question resides in what risk factors are not being considered when choosing the best screening option. Age is one of the best documented risk factors, with the incidence of BC being extremely low before the age of 30 and having a linearly increase until the age of 80 [7]. Body Mass Index has also been shown to be a potential risk factor for the development of BC but only in post-menopausal ages [7,8]. Prior history of neoplastic or hyperplastic breast disease also presents itself as a risk factor for the development of BC. When it comes to family history, a woman who had a first-degree relative with BC when was 50 years or older, is almost twice at risk of developing breast cancer than a woman with no family history of BC [7]. Early menarche, late first full-term pregnancy and late menopause are three major risk factors for breast cancer [9]. Normally, the earlier the age of the first menarche, the higher the cancer risk. The fact that both women with early menarche and later menopause are at higher risk of BC, can lead to the conclusion that prolonged exposure to estrogen is also a risk factor for this disease [9]. Longer duration of the breastfeeding period is associated with a diminished risk of breast cancer, in comparison with women that had shorter breastfeeding periods. Use of oral contraceptives also puts women at higher risk of developing BC [10]. As it was previously discussed, the existence of the BRCA1/BRCA2 mutated gene in women karyotype puts them at higher risk of BC, compared to women who do not possess that gene [11]. Besides these risk factors, in 1976 Wolfe, started studying the association between breast parenchyma patterns and breast cancer. Wolfe showed that a prominent duct pattern helps to classify a woman as having higher risk than average for developing breast cancer. Wolfe also stated that it is possible to predict which women will develop breast cancer and which are less likely to develop it based only on the parenchymal pattern [12,13,14,15].

Many descriptors of these texture patterns have been documented. Mammographic density is one of those descriptors, normally represented numerically by percent mammographic density (% PD), that is also highly associated with an increased risk of breast cancer [16,17,18]. In fact, women with 60–70% PD are at four to five times higher risk than women with fatty breasts. Dense breasts are not only at higher risk of developing breast cancer as are also more prone to more aggressive tumors.

Screening programs all around the world use mammography, that can be acquired in a cranio-caudal (CC) and/or in a mediolateral-oblique (MLO) view, as a standard method for diagnosis, but although widely used, mammography has both benefits and harms. The aim for an early detection of this disease started in the beginning of the 20th century with awareness campaigns, but a decrease in BC mortality was only observed when the first mammographic screening started. On the bright side of mammography screening, life-threatening cancers will be detected early, improving prognosis, and consequently, decreasing risk of mortality. Studies point out that BC mortality rates, decreased at least 20% [19] thanks to an increase in mammographic screening—some studies even point out a reduction ranging from 30–50% [20]. Besides that, since cancer can be detected in an early stage, the available treatment can be less invasive and, consequently, have lower costs. The treatment will also be less intense, resulting in fewer time off of work, and, consequently, smaller money losses.

One of the problems associated with mammography is the rate of false positives. In Europe, the risk of having a false-positive result, for women in the range of 50–69 years having biennial screening, is about 20%. More alarming are the results in the United States, where all screened women will experience one false-positive in their life. These false-positive results have an impact in women lives, especially in day-to-day well-being and in costs concerning healthcare. But the presence of false positive is not the only downside of mammography. A summary of the benefits and harms of mammography in 1000 women with a screening every two years showed that 200 of them will experience a false positive, 30 will have a biopsy due to the false positive result, 15 will be over-diagnosed and three will develop interval cancers – the name given to a cancer that appears between two consecutive mammograms. These interval cancers may have been developed between the two mammograms, however, around 35% of them were already present in the previous mammogram but were overlooked. This means that the patient received a false negative result that can occur because, in mammography, there is an overlap of tissue that can obscure the presence of cancers [21]. Since the population being screened is mainly composed of asymptomatic women, it is expected that with increased screening, it will also be seen an increase in cancer incidence. Life-threatening cancers will be detected early, improving prognosis, which is clearly a point in favor of mammography screening, however, cancers that would never be detected and that, in theory, were not harmful for the woman who presents it, will also be diagnosed. This is called overdiagnosis. Overdiagnosis leads to an ethical dilemma since there is a probability for the patient to live longer with cancer than with the treatment, and this decision-making process could lead to an increased anxiety state of the patient [21]. Another important aspect to consider, related to this type of screening, is the relation between mammography and dense breasts. The sensitivity of mammography decreases in women who have dense breasts (30–64% vs. 76–98% in women with fatty breasts) [19], which occurs because cancers have attenuation coefficients closer to dense tissue. Actually, a study from 1999 [22] showed that there was a significant trend between breast density and the appearance of false positives. Since it is known that breast density is an important risk factor for the development of BC, the fact that mammography does not perform so well in dense breasts should be of great concern. As seen, there are multiple downsides to mammography and, yet it continues to be the standardized screening method. However, in 2014, the Swiss Medical Board stated that the harms produced by these screening programs outweighed the benefits and, therefore, they recommended Switzerland to stop all the mammography screening programs [23].

New technologies that allow a risk stratification, in line with the current medicine paradigm of preventive and personalized care, could help overcoming some discussed problems associated with the screening programs.

A review of Artificial Intelligence (AI) in the field of Breast imaging was already performed by Le and his colleagues [24]. In this work, a brief introduction to Artificial Intelligence is performed, concerning commonly used terminology and widely used algorithms. Applications of computer aided detection (CAD) systems in mammography screening are explored, like the automatic detection of breast cancer, or the distinction between malignant and benign lesions. Software based on AI for breast density classification assessment is also addressed. The authors, beside describing deep learning approaches in mammography, proceed to address relationships and applications of AI to digital breast tomosynthesis, ultrasound and MRI. Finally, the implementation of AI-CAD systems in clinical practice, the limitations of these systems, obstacles to its implementation and future applications are discussed.

In the current work, Section 2 explains the methods by which this review was performed in terms of inclusion criteria. Section 3 presents the results of this review, with each included paper being analyzed in terms of proposed goals, methodology used, and results obtained. Finally, in Section 4 a conclusion and a discussion about future endeavors is made.

## 2. Methods

The review done here aims to present a global picture in what is already done in the field of breast cancer risk assessment through computerized methods, using mammograms. A search in Google Scholar using different Boolean operators and the keywords—breast cancer risk, mammography, machine learning (ML), features, parenchyma/texture patterns—was performed. This search produced eight-hundred and forty-two matches that were screened through title and/or abstract. In order to be considered for this review, papers should meet the following inclusion criteria:

(1) Aim for a risk assessment, either by differentiating risk groups, predicting a risk value, or proposing new methods for the said assessment, using Machine/Deep Learning (DL) tools.
(2) The methodology used should consider textural features with/without epidemiological factors.
(3) Mammography images should be used for feature extraction, when that procedure is done.
(4) All papers’ publication date should be within the 2000–2020 range.

**Figure 1:**
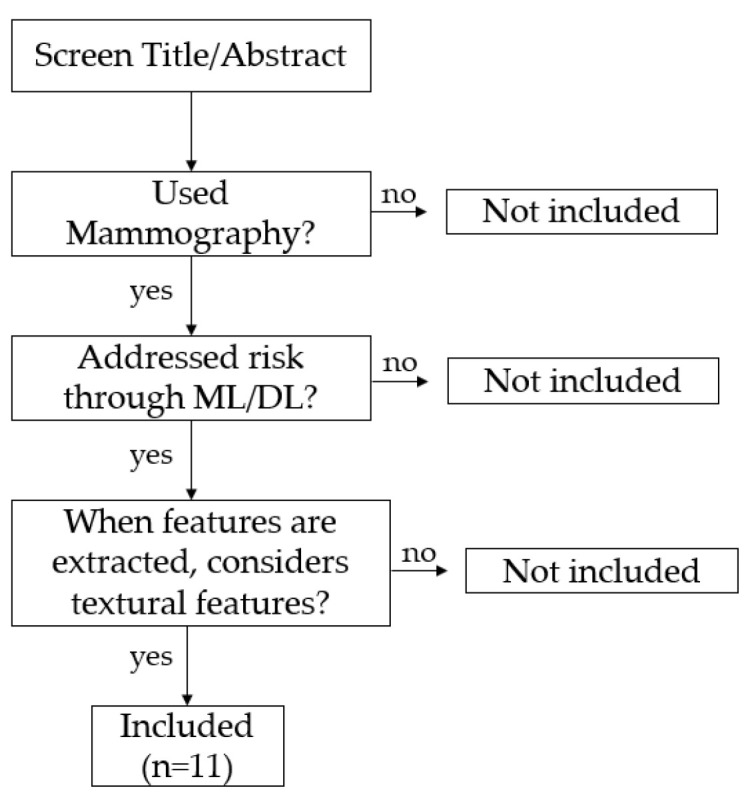
Paper Inclusion flowchart, for a search within the range 2000–2020 for publication date.

Papers cited by the accepted manuscripts were also screened through the previously referred criteria. Only the articles that better served the scope of this work were considered, which resulted in 11 included manuscripts. Figure 1 represents a flowchart of the methodology used. 

## 3. Results

### 3.1. Risk Assessment Using a Single Region of Interest

In the start of the millennium, Huo et al. [18], investigated feature selection in breast parenchymal patterns, for BC risk assessment. The specific aim of the study was to classify women, based on their mammograms, into high or low-risk groups. To label the training data, the authors used Gail’s model, which asks as input some epidemiologic information: age; age at the first menarche; age at full first-time birth; number of first-degree relatives with BC; and number of previous breast biopsies; then, with that information, calculates a probability for developing BC [25]. This method, although very used, has its limitations, for example, it cannot be used in younger women, and it is unable to predict risk for women with the BRCA1/BRCA2 gene.

To be included in the low-risk group, besides having a risk lower than 10%, women could not have any family history of BC. Mammograms from women with the BRCA1/2 mutation were considered high-risk. An important step in dividing the dataset needs to be referred, as it happens in other similar researches. The age of mutation carriers and the “low-risk” group tends to be different, and, in order to avoid bias due to that difference, an aged-match dataset was constructed, and risk analysis was also conducted in this “sub-dataset”.

Once the datasets were divided, mammograms proceeded to be pre-processed feature extraction being conducted in a pre-defined region of interest (ROI). Intensity-based features, statistical measures based in absolute pixel value; co-occurrence (GLCM) features [26], metrics that describe pixel pairs co-occurrences throughout the image; and two Fourier analysis features were extracted—a description of these features can be found in the referred manuscript. Once the extraction step was done, each feature was analyzed, through receiver operating characteristic (ROC) analysis, in order to access their discriminative capacity between high-risk and low-risk groups. The area under the curve (AUC) for each individual feature ranged from 0.53 ± 0.09 (minimum gray-level) to 0.87 ± 0.05 (skewness). After this evaluation, a feature selection method was applied so as to reduce the dimensionality of the problem and increase computational efficiency. This was achieved using stepwise selection followed by linear discriminant analysis. Discriminant Analysis chose intensity-based and co-occurrence features for the best set of features in the task of differentiating the two considered risk groups. Curiously, the chosen features were the ones that presented better discriminative capacity in the individual ROC analysis for the age-matched group. The linear discriminant analysis approach presented an AUC higher than any of the features alone—0.91.

Besides proving the usefulness of the texture features to characterize the difference between low and high-risk women, some interesting conclusions can made from this study considering features’ average values for each group: the textural patterns from high-risk women tend to be coarser and lower in contrast; skewness measure should have negative values for high-risk women; and all the remaining intensity-based features should have higher values for high-risk women.

To ensure that a good parenchyma characterization could be done through one projection, the authors made a correlation study between CC-L and MLO-L, and between CC-L and CC-R views for each feature, which provided positive results. A limitation of this study might also be the fact that some of the low-risk women may have the mutation without knowing, which clearly affects features’ discriminative capacity.

**Figure 2:**
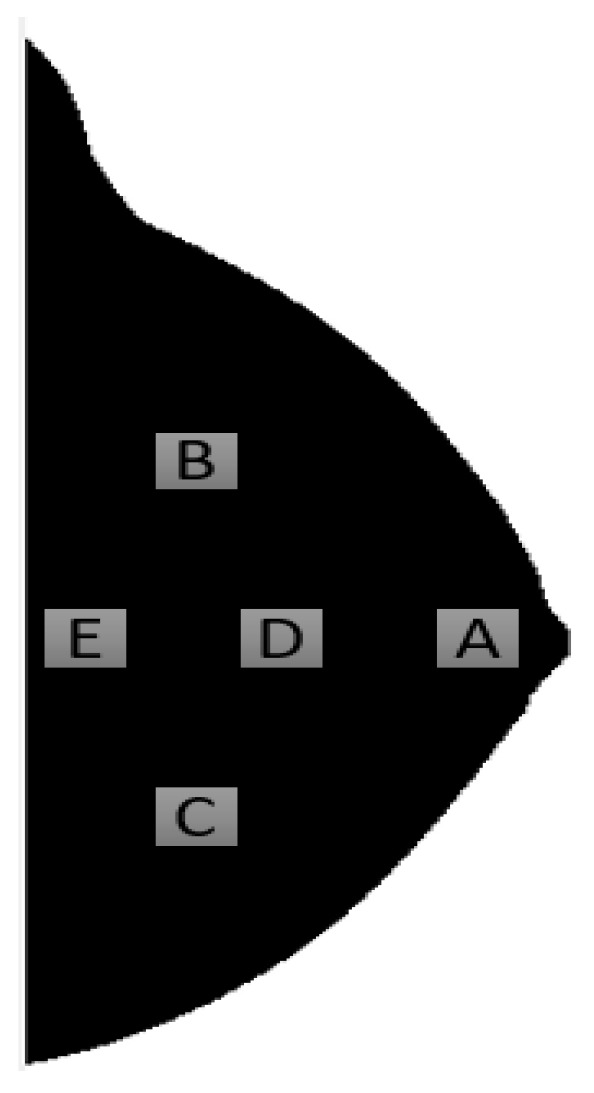
Different locations (**A**–**E**) where features were extracted for ROI evaluation.

Li et al. [27] studied, in 2004, the effect of ROI size and location for feature extraction in BC risk analysis. The performance of the size and location of each region was evaluated in the task of differentiating high-risk (mutation carriers) and low-risk women. An aged-matched group between mutation carriers and low-risk women was created and used for risk analysis in this study. Researchers designated five different ROI locations, as depicted in Figure 2, identified by the letters A, B, C, D and E.

For size analysis, 2 ROI’s, one with a medium and other with a small size, were directly defined in the center of the larger, pre-defined, ROI, at locations A, B and C. The extracted features were the same that Huo extracted with the addition of a fractal dimension measure [28]. Stepwise feature selection with LDA was employed as a feature selection method, after feature extraction from the different ROI’s was performed. Each feature was individually evaluated for its discriminative capacity between the considered groups, through ROC analysis, and the same was done to the linear discriminant analysis approach. Descriptors from Fourier analysis, co-occurrence, intensity-based and fractal dimension were chosen by the feature selection methodology, a process that is fully described in the paper and complemented in [29].

In what concerns to ROI size, for location A, the AUC for each individual feature ranged from 0.68 to 0.83 with the lower results being associated to smaller regions of interest. The performance of the LDA approach was of 0.92 in the original ROI, with AUC’s of 0.87 and 0.89 in the medium and smaller ROI’s, respectively. In the size analysis, significance was only achieved for one feature and for the LDA approach between the large and medium ROI and for the fractal dimension between the large and small ROI. The values of the AUC in the LDA approach for region B were substantially lower than the ones observed for region A and, besides that, no statistical significance was observed for this region neither in individual features assessment nor in the LDA approach. Finally, for region C, the only statistical significance was achieved for feature contrast, between large and medium, nonetheless, no statistical significance was obtained in the discriminative capacity between different sized ROI’s.

Analyzing ROI location effects, for comparison purposes, only the features selected by the LDA approach for region A were considered. Most of the individual features were statistically significant different between different locations, and the same can be said about the LDA approach that presented AUC’s of 0.92, 0.78, 0.69, 0.84, and 0.79 respectively for each region. A statistically significant decrease was observed in the LDA performance if the ROI was moved away from region A, that is located immediately behind the nipple, which probably explains why most of the approaches in this area of research use this location for feature extraction. The authors point out that the fact that the region immediately behind nipples has the best discriminative capacity may be due to the existence of a dense component in that breast location. The group still states that, in the future, research should extract features from the entire breast and compare the results with the ones obtained with a single-ROI approach that are, besides its limitations, positive.

In 2005, Li et al. [30] aimed to prove the usefulness of breast parenchymal patterns present on mammograms in the field of breast cancer risk assessment. As it had happened in the previous analyzed researches, the authors aimed to extract texture feature from mammograms to differentiate high-risk women, mutation carriers, from those who have a low-risk of developing the disease. In order to be considered for the low-risk group, besides the two conditions presented in the research done by Huo in 2000, women could not had been diagnosed with breast cancer in the past and, if they had done a biopsy, they were not considered. Once again, since age is an important risk factor, an age-matched group between low-risk women and mutation carriers was created. At each mammogram, features were extracted from the pre-defined ROI and, although ranging the common groups, they slightly differ from previous researches. Besides intensity-based, co-occurrence, Fourier and fractal dimension features, mean gradient, an edge frequency feature that measures the coarseness of a surface, was part of the studied features and is described in the paper. ROC analysis was used to evaluate the individual performance of each feature in the task of differentiating high-risk and low-risk women. The results ranged from 0.66 ± 0.05 (Entropy) to 0.86 ± 0.03 (co-occurrence contrast) in the entire dataset, and from 0.67 ± 0.05 to 0.86 ± 0.05 in the age-matched group, with statistical significance (*p*-value < 0.001) being achieved for all features. The authors proceed to present a figure where a distribution of skewness measure in the population can be observed. From that, it is drawn the conclusion that high-risk women present negative values of skewness, as advanced by Huo in 2000, since these women normally have denser breasts, when compared to lower-risk women, and, in general, high-risk women present lower skewness values than women at the low-risk group. Contrast helps to describe the local tissue variation, and higher values of these features were observed in low-risk women, which leads to the assumption that mutation carriers tend to present texture patterns low in contrast. Besides that, results analysis in terms of feature values lead the authors to state that mutation carriers tend to have coarser textures than low-risk women. Although these conclusions from contrast and coarseness can be made in general, not all women follow this trend. Nonetheless, this study presents itself as another proof that mammographic texture patterns can be successfully used in the field of breast cancer risk assessment.

### 3.2. A Disruption from the Classical One-ROI Approach for Risk Assessment

Another interesting research is the one done by Tan et al. [31], where the authors aimed to evaluate the viability of predicting BC risk in women after they had a negative mammogram. Given a sample of screened women, each woman was considered for the study if had had two consecutive mammograms acquired in the authors’ facilities and if the first mammography was negative. A dataset was then created with the accepted women, where each case was composed by two mammograms—defined as “prior” and “current” evaluations—and based on the current evaluation, the dataset was divided into three subgroups. The first was composed of women who had positive results, confirmed with other evaluation methods, and women who had pre-cancerous masses that were removed. The second subgroup consisted of women who had abnormalities in their mammograms, were recalled, but then the lesions proved to be benign. Finally, the third subgroup included women with negative mammograms and that were not recalled. In the study, the researchers used all the “prior” evaluations, that were negative, to assess breast cancer risk in the “current” evaluation. It is important to mention that, for each dataset case, age, family history of BC, and the density rating by the BI-RADS scale were the epidemiologic risk factors considered. For feature analysis purposes, the authors segmented the breast in different regions and extracted features in the segmented areas, therefore considering the whole breast for feature extraction. The extracted features could be divided, once again, in different subgroups: Intensity-based and co-occurrence features—in the horizontal direction—were extracted. Run-length (RL) features [32], that describe runs of same intensity pixels in a given image were also considered in both the vertical and horizontal direction. Besides that, another group of features, that the authors called “x-axis/y-axis histogram cumulative projection” was considered, a brief explanation of this group of features is given in the paper. Features were computed in the entire breast and also in dense breast regions, that are defined as regions that are composed of pixels with intensity above the median value of the whole breast.

Although the previously referred features were computed, they will not be directly used for risk assessment purposes. What is done is that each feature is calculated from the CC view of each breast and then, features that describe the bilateral asymmetry of each individual feature will be computed through the following equations:

(1) $$F_{Assymetry\;1-60}=\frac{\left|f_{i}-g_{i}\right|}{max\left(f_{i},g_{i}\right)}$$

(2) $$F_{Assymetry\;61-120}=\left|f_{i}-g_{i}\right|$$

(3) $$F_{Assymetry\;121-180}={\left|f_{i}-g_{i}\right|}^{3}$$

A set of 180 asymmetry features were calculated and, adding the epidemiologic data, a final set of 183 features was considered. To choose the best features, a forward floating selection method was applied- proposed and described by [33]. Once the selected features were retrieved, a support vector machine (SVM) classifier, with a radial basis function kernel, was trained and tested with the referred dataset.

Classifier’s performance validation was done using a 10-fold cross-validation methodology and, at each testing step the algorithm outputted a score ranging from 0 to 1. The higher the score, the higher the probability of having an “image-detectable” cancer in the next screening. Feature selection methodology, besides age, selected features from Run-length, Intensity-based, and cumulative projection groups. For classification purposes, using only the first and third subgroups the classifier had an AUC of 0.716 ± 0.020. Considering all the cases, the SVM model correctly predicted 71.3% using a 0.5 score as a decision threshold for a classification between negative/benign cases and positive cases. Some limitations of the developed work must be considered: The fact that the dataset used was produced in laboratory does not reflect the ratio between positive and negative cases in common BC screening programs; the methods used for validation may have resulted in bias and, the fact that the same portion of the dataset was used both for features selection and to evaluate the classifier accuracy may also have resulted in some bias in the process of optimizing the algorithm. Besides that, only asymmetry features were computed, which could lead to some masking effects of the effective texture of the parenchyma.

Zheng et al. [34] advocated, in 2015, that approaches that use a single ROI for risk assessment are insufficient since they cannot properly define all breast parenchyma, since it does not consider its heterogeneity. The authors stated that texture characterization should be done across all breasts, using structuring elements for feature extraction. The idea that these descriptors, calculated across all breasts recurring to structural elements, could improve texture description was advanced by these researchers and that resulted in the development of a software, that they call lattice-based approach, to extract features from structural elements across the entire breast. A comparison of the association of texture features with cancer between the lattice-based approach and the single-ROI methodology was performed. To use this methodology, breast area definition and pixel value normalization was performed. The next step was PD% computing, achieved by using a clustering algorithm that subdivided the breast into different regions with each region having approximately the same composition; then, a SVM algorithm would classify each subregion as being “fatty” or “dense”. PD% was simply computed by dividing the number of dense areas by the total number of subdivisions defined.

The clustering algorithm used here is a variation of the fuzzy c-means (FCM), which works by giving each pixel/data point a membership degree to each cluster. This degree is related to distance metrics taken between the point and the cluster: the lower the distance, the higher the membership degree. Fuzzy c-means will then, through various iterations, try to minimize the intra-cluster variance while maximizing the inter-cluster variance. Besides breast segmentation for % PD calculation, this algorithm has been used for other purposes. A group of researchers used a variation of the FCM, where the influence of spatial neighbor pixels and similar super-pixels is incorporated in the model, for lesion segmentation on brain and breast MRI as also in mammograms [35]. The idea of modifying this algorithm was held by the fact of FCM being highly sensitive to noise because spatial information was not considered. The experimental results were evaluated with different metrics—specificity, accuracy and false alarm Rate (FAR)—and compared to other commonly used segmentation algorithms. Breast MRIs and mammograms were used to assess lesion segmentation, while brain MRI is used to evaluate algorithm’s performance in noisy image enhancement. For the brain MRI image, the highest results for both accuracy and specificity were obtained for the methodology proposed by these authors, and the same can be said about the lowest FAR results, proving that best results are obtained by this methodology. Moreover, it also shows that the noise problem can be countered with this algorithm. Different types of breast tissue, breast size and tumor size were considered when studying segmentation of breast MRI images. Their results show that the standard FCM methodology achieved poor results, due to noise, while their methodology provide the best results, with tumor edge being as clear as possible (and not blurred as it happens with other algorithms). In terms of accuracy, specificity and FAR, the proposed method has the best results across all cases. Finally, for tumor segmentation in mammograms, four cases were analyzed, with different characteristics, and the results show that the methodology adopted by the authors was the one that was closer to the standard results obtained by clinicians/experts. Once again, accuracy, specificity and FAR achieved their best results for the authors’ methodology. This study provided clues that the proposed methodology can outperform commonly used algorithms in the task of lesion or organ segmentation, even in the presence of noise.

Still concerning brain MRI, FCM has the potential to be used in a pipeline related to neuro-radiosurgery [36]. The authors that propose this approach relate that assessing necrotic tissue that occurs within the tumor might add knowledge about tumor development. The goal of the methodology proposed is then to use FCM for necrosis extraction, after a gross tumor volume segmentation(GTV). This pipeline might allow, for example, to selectively choose the given dose accordingly to zone resistance to radiation. The use of FCM after GTV will make the tumor characterization more precise, with necrotic and enhancement areas being distinguished—by clustering them. This brain tumor necrosis extraction will provide an increased clinically valuable insight about cancer characteristics, while playing an important role in neuro-radiosurgery, in terms of dose redistribution. Several metrics, ones related to spatial overlap, and others concerned with distance were calculated. The first (sensitivity, specificity, etc.) compared the regions that were achieved with this methodology, against the segmented areas obtained by an expert. The latter, contrary to overlap-based metrics, considers the boundary’s voxel position in the space, which should be used, since boundary delineation is very important in radiosurgery or treatment planning. Considering overlap metrics, the proposed method provides higher results than conventional methodologies, providing clues that this is in fact an accurate and reliable method. These positive results are corroborated by the spatial metrics, which indicates that this pipeline serves its initial purposes. Given that, FCM presents itself once again as a good clustering algorithm for different goals.

Getting back to Zheng’s research, once % PD calculation was performed, feature extraction could be conducted, and for that, the lattice-based approach needed to be considered. The authors display a grid over the entire breast tissue, where different values for the distance between each intersection point, *D*, and for structural element size, *W*, can be considered. The structural elements are centered in the intersection points and will serve as different ROIs for feature extraction, so, each computed feature will have different values across the breast. Although the optimal values for *D* and *W* might be different for different regions across the breast, authors considered a fixed and equal value for these components, resulting in a breast that is coated with structural elements. Intensity-based; co-occurrence; run-length; local binary pattern; fractal dimension, and structural features that describe “flow-like structures within the breast” were also considered—the authors provide references for these novel features. In order to look for the optimal W value, approaches with three increasing sizes—small, medium and large—were tested. Each “final value” of the features was defined as the mean value of the said feature across all structuring elements and the association between the computed features and breast cancer was evaluated with a logistic regression classifier with leave-one-out cross validation. Univariate and multivariate analysis were conducted, with feature selection being done in the later one through a forward feature selection methodology applied at each cross-validation loop. Considering univariate analysis and taking all window sizes into account, the average AUC over the GLCM features was of 0.58 ± 0.03, which is better than the one presented by the intensity-based features, being of 0.56 ± 0.05, the same value that was presented by the run-length features. Structural features presented a worst AUC than GLCM features, with a value of 0.57 ± 0.06. Comparing window sizes, the performance seems to be better for small *W* values, with an average AUC of 0.58 ± 0.07 for a small window against AUCs of 0.57 ± 0.05 and 0.54 ± 0.03 for sizes medium and large, respectively. The feature that presented a higher discriminative capacity was fractal dimension for sizes small and medium, presenting an AUC of 0.69 ± 0.03. In where it comes to multivariate analysis, using a logistic regression, the performance was also better with smaller *W* sizes and the AUCs values obtained were of 0.85 ± 0.02, 0.81 ± 0.02 and 0.76 ± 0.03 for sizes small, medium and large respectively. All the features outperformed PD% performance and no significance was obtained in the model when PD% was added to the set of features. The lattice-based approach significantly outperformed the single-ROI approach either from the retroaerolar area (AUC = 0.60 ± 0.03) or the central breast region (AUC = 0.74 ± 0.03), despite the *W* size considered. The results may cause some surprise once, contrary to what was proven by Li in 2004, the central breast region ROI performed better than the retroaerolar area. Given what was discussed about this topic, some conclusion must be drawn: the extraction of features by itself does not result in better discriminative capacity but it is the combination of those features that gives positive results; and *W* size is important for a better discriminative capacity, with an approach that considers smaller *W’s* providing better outcomes. Nonetheless, some problems related to the work done here must also be considered: the use of equal values for *W* and *D* might be a limitation, since much more combinations could be tested if that condition was not present, what could result in a better discriminative capacity; and PD% calculation was done by considering one of the many possible options available to perform that computation, what could also bias the results.

Changes in mammography texture features for breast cancer risk assessment were studied by Tan et al. [37] in a study where, as done in 2013, the authors conjectured that features that describe bilateral asymmetry might be important markers to predict near-term breast cancer risk. What is done differently here is that the authors aim to found features that allow the prediction models to have a better performance, and they compare the risk scores generated by their model with a time-lapse between a negative and positive mammogram of a patient with a number of sequential mammograms. For this study, women with at least four sequential mammograms were considered, with the cancer/risk cases being the ones that were diagnosed with breast cancer in the most recent mammogram, and the remaining being considered as control. The most recent mammograms were considered as “current” evaluations and all the previous mammograms were considered “prior” evaluations, classified as negative in an evaluation done by radiologists, with no recalls happening in the “prior” group. The authors provide an extensive description of two used groups of features—structural similarity features and Weber descriptors—that will not be replicated here. Besides those groups, GLCM, RL and intensity-based features were likewise computed. For GLCM and RL features, only the mean value and the maximum value of each feature across computation directions (0°, 45°, 90°, and 135°) were considered. The breast was segmented and the ratios of the area, within the segmentation, with intensity values above mean pixel intensity values for the whole segmented breast, were considered for PD% measures. Concretely, the authors used three thresholds to compute this ratio: (a) values above the mean; (b) values above the maximum; (c) values below the minimum. Once this was achieved, the study proceeded with the calculation of four features based in % region cutoff of the density function. Using a Sobel gradient operator, statistical measures driven from gradients were considered. Finally, the difference between the number of pixels present in each breast for the same patient was calculated. Equation (3) was used, and the result represented the bilateral asymmetry features between the left and right breast. A SVM algorithm, with a linear kernel, was trained and tested using a leave-one-out cross-validation methodology and, at each training session, stepwise regression was used to select the most relevant features. This procedure was done three times, one for each of the “prior” mammograms. Besides evaluating risk score evolution across the three “prior” mammograms, the authors also aimed to study variations of individual feature values between groups, what was done by computing the mean and standard deviation of the features between the “negative” and “positive” group for each “prior image”. After that, using a t-test, *p*-values that assessed the difference between groups at each “prior image” were generated. Given the already explored problem of significantly different ages between high and low risk women, the authors repeated the SVM procedure with two different age-matched groups using a criteria of ±1 year and a criteria of ±3 years. Apart from that, the authors trained and tested three different classifiers and, at each time, they used the features selected through one of the prior images sets. Concerning the results, the AUC increased as the time approached the current evaluation, with the values being 0.666 ± 0.029, 0.710 ± 0.028, and 0.730 ± 0.027. As for feature difference results, different trends can be observed, with features having higher discriminatory capacity across the three “prior” examinations (structural similarity), others having significant discriminatory capacity in one or two of the examinations (run-length), and others with no discriminatory capacity in any of the mammograms (contrast). In line with previous research, one can conclude that although individual features might have good discriminatory capacities, it is the use of a multi-feature ensemble, recurring to a machine learning algorithm, that allows a good breast cancer risk assessment. Considering the predicted risks and defining the midpoint as a threshold, the SVMs had an accuracy of 65.7%, presenting a sensitivity of 46.5% and a specificity of 83.0%. When considering the algorithms trained and tested with the age-matched group, no significant difference in AUCs was observed. This, and the 2013 study are approaches widely different from the common ones since they add time-dependent variables for risk prediction that can be used to develop novel techniques for risk assessment in a personalized fashion. The authors proved a decreasing trend in AUC values from the most recent “prior” evaluation to the oldest but got results that point to the fact that this decrease might not occur linearly.

In this research the authors aimed to avoid the 2013 limitation of the cases not representing a screened population by ensuring that the cases were randomly selected by people who were not involved in model construction, what made the average women age in the “positive” group to be higher than the average in the “negative group” mimicking what happens in screening programs. When analyzing this study, some limitations must be considered: (a) model reproducibility might be affected by different acquisition systems and noise and, therefore, methodologies to reduce acquisition impact must be developed; (b) image features related to local region bilateral asymmetry were not used and might improve the obtained AUC; (c) this model does not include epidemiological/ risk factors which is a flaw, when compared to existing models; (d) the low accuracy values for individual features might be an obstacle to clinical use; (e) an examination of how features varied across the prior examinations was not considered but might be an interesting line of research to pursue.

In 2019, a group of researchers [38] aimed for a novel approach for breast cancer risk assessment. In this study each cancer case had age, ethnicity, and BMI matched controls. In what comes to ethnicity, all cases were correctly matched, 83% of the cases were matched for age (±5 years) and 94% of the BMI cases were also correctly matched (±1.5 kg/$m^{2}$). Feature extraction and PD% (done with the Volpara software, Lynnwood, WA, USA) were extracted from the CC view and, for cancer cases, contralateral images were used to assess risk. For feature extraction, five different subgroups of features can be considered: Intensity-based; GLCM; run-length; structural patterns, like LBP and fractal dimension measures; Weber local descriptors; Sobel gradient approaches introduced in the previous articles; and a new set of features called MRELBP, that can describe macro and microstructure information, having low effort computation and that are robust to image noise; and finally, spectral features, related to Gabor, were also computed. Model validation was done with leave-one-out cross-validation. Stepwise regression was used for feature selection and, at each iteration, F-statistic was calculated in order to assess if each feature had a statistically significant contribute to the model. Spearman’s rank correlation was also computed to check for correlation between the more commonly selected features at each leave-one-out loop. After feature selection was conducted, the selected features were merged using linear discriminant analysis with the LDA classifier producing a risk score of each case to have breast cancer, meaning that, at each leave-one-out step, 500 risk scores were generated. When comparing the mean risk scores between cancers and controls, the system output a risk of 0.55 for cancer cases and of 0.44 for controls and this difference was statistically significant (*p* < 0.001). The same cannot be said about PD%, that was of 16.7% for cancer cases and of 16.2% for controls, but with a *p*-value of 0.50. The LDA classifier provided an AUC of 0.68 (95% CI 0.64–0.73), while the Volpara methodology presented an AUC of only 0.52 (95% CI 0.47–0.57), this difference was tested and achieved statistical significancy, proving that the classifier is able to extract more useful information than the measures of PD% done by the software. Intensity-based, co-occurrence, gradient and MRELBP features were amongst the chosen ones by feature selection. The six selected features were not all correlated, and some were only correlated to another two selected features, which proves, by the relative positive results obtained in the discrimination between cases and controls, that the LDA classifier could combine information from both correlated and uncorrelated features. Nonetheless, it can be noted that the obtained AUC was relatively low when compared to other studies and the authors pointed that this might happen due to differences in age and ethnicity of the women used in this study. Nonetheless, this study provides a proof that the methodology used for risk stratification in Caucasian women can be used, here, in Asian population at the same time that also provides new features that have a great discriminative capacity in what concerns to breast cancer risk assessment.

Still referring to Asian population, Gandmokar et al. [39], in the Fifteenth International Workshop on Breast Imaging (Leuven, Belgium, 2020), presented a breast cancer risk prediction model based not only in mammographic texture feature but also in an enormous set of epidemiological features (or risk factors), categorizing women into high-risk and low-risk groups. For each woman in the study the following epidemiologic factors were obtained: height, weight, BMI, age at menarche, menopause status, age at menopause, age at first delivery, parity history, number of children, breastfeeding history, personal history of breast cancer, family history of breast cancer, and degree of consanguinity. For feature assessment, contralateral images from the cancer patients were used and considered the high-risk group and control women images were labeled as “low-risk”, it should be noted than only CC views were used. Breast segmentation for density calculations was done using a software called AutoDensity [40], from which results two thresholds, the first that represents the bright area of the mammogram and the second, since that is computed based on the dense area, represents the brightest area. Features concerning intensity-based groups, GLCM manipulation, and Fractal Dimension were extracted (from the bright and the brightest area) and added to the epidemiologic set of features. Then, these features were fed to an ensemble of decision trees, acoplated with AdaBoost that was validated with a leave-one-out cross-validation methodology and presented an AUC of 0.884 (CI 0.838–0.913) in differentiating risk groups. Although the results are promising, study limitation must be assessed; (a) the model was validated in a small dataset; (b) contralateral images were used as high-risk but since the goal is to do a risk prediction the model should be constructed using prior mammograms; (c) study population was from women recruited from a single city which does not represent the usually found differences between women from different locations; (d) the control and cancer cases were driven from different datasets.

### 3.3. Deep Learning in Risk Analysis

Deep learning, a sub-field of machine learning that can learn directly from a raw input, is also used for breast cancer risk prediction. In 2016, Kallenberg et al. [41] aimed to use unsupervised deep learning to perform breast density segmentation and mammographic risk scoring. In order to overcome that problem, this research uses deep learning methods to learn features from mammograms, in an automated fashion. The DL model used is called convolutional sparse autoencoder. An autoencoder can be understood, in general, as a neural network that works towards the aim of learning the input so well that will also learn to replicate it as the output of the model; the process by which this occurs is based in the learning of how to correctly compress and encode the input that will ultimately be reconstructed. An autoencoder has an encoder, that maps the input layer to the hidden layer, and a decoder, that maps the hidden layer to the output layer. Once the features are learned and extracted, the resulting set of descriptors will be used to associate the data with previously defined labels. This model was applied in two distinct phases; first, it was asked to the model to make breast segmentation based on density values, and second to address mammographic parenchymal patterns, considering the goal of predicting future breast cancer development. The methods used here are based in a denoising autoencoder, an approach in where the hidden layers have a higher dimension than the input layer. The ground base idea is that the encoder will receive a corrupted version of the data and will then learn how to reconstruct a version of the data that is not corrupted [42]. What also happens in this methodology, is that various autoencoders can be assembled together so that the learned features increase progressively in level of abstraction. The process by which this occurs makes features to be learned by one encoder, with the respective decoder being removed but the features being kept, then, the processed data is passed through a new autoencoder, where data is reconstructed. This process occurs until the reconstruction of the last hidden layer occurs [42]. The goal here is not to extract specific features, but rather to learn features directly from the mammograms, hoping that this methodology will be highly generalized, in opposite to what happens, in general, to a manual extraction approach. The models are trained in a forward propagation model, with a constant update of the learned weights, in order to optimize the process. A way of optimizing the features is to look for a minimization of the difference (or loss) between the predictions of the “top most layer” and the real labels. A division into multiple layers is done for feature learning, before a classifier is trained to make prediction in the “top most layer”. This results in a “multioptimization” problem, that the authors point to have some advantages, like the fact that features are learned faster and in a more secure way, since each layer is specifically optimized, or the fact that these methodologies can incorporate other units, like classifiers, that can be independently optimized. In this case, the authors use a sparse autoencoder, which is a regular autoencoder where a sparsity limitation was forced in the hidden layers [43], for learning features that represent information at multiple scopes. The goal is to predict a “label mask” to each image, and, not only the entire image cannot be computationally used to retrieve label masks, as downsampling the data is also not possible since important information could be lost. What is suggested, instead, is that the algorithm should learn local neighborhood regions in an image-patches. Concerning the notation used in the paper, the goal is to map a patch, $x\in X={\mathbb{R}}^{c\times m\times m}$, with *m* × *m* being the size of the patch and *c* being the number of channels in the patch, to a label patch, $y\in Y={\mathbb{R}}^{C\times M\times M}$, with *M* × *M* being the size of the patch and having one channel per label. Although the image and the label patches may have different sizes, they are centered in the same location. Then, for training purposes, training data will be used to map X to Y. The training data consists in (x,y) pairs extracted from random locations across the images that are concerned with this part of the work. The mapping from X to Y does not occur directly, what happens is that abstract feature representations are learned across multiple layers. In other words, the input enters the algorithm and crosses multiple layers, with the output of one layer being the input of the next, where multiple transformations are made and learning is performed, until the last layer is reached, and a final feature representation is obtained. Finally, a classifier will be used to map the final feature representation to Y. For testing purposes, the hypothesis that was trained will be applied to a new image in all possible patches within the said image—using a sliding window. When doing this, a problem can arise: if the predicted output region is bigger than a pixel, there are predictions that may overlap. The problem is solved by calculating the average probability for each class. Mammogram analysis, conducted in a multi-scale fashion, is done by applying the discrete scale space theory, through a Fourier implementation. Algorithm unsupervised architecture consists of four layers: a convolutional layer, a pooling layer, and two final convolutional layers. Going deeper in convolutional architecture, what happens is that the convolutional layer will receive the input data, convolve it, do some transformations, and then send the results as a non-linear activation function to create an activation/features map. The output of the layer can be fed to another convolutional layer or to a pooling layer. The pooling layer was defined based on the goal of the study, once it is invariant to small distortions, but it is highly sensitive to small-scale details. Features are learned for each scale alone and only merged after the learning process.

The approach proposed by the authors aims for an overcomplete feature representation, which means that this representation is larger than the input, and resorts to the concept of sparsity. Sparsity can be used in feature representation by: (1) forcing most of the entries to be zero and leaving few non-zero entries to represent the input signal; (2) narrowing the number of examples that activate each unit. In this work, both approaches are combined, leading to, respectively, “a compact encoding per example” and to “example specific features”. Sparse overcomplete approach is robust to noise and, since each example is going to be represent by specialized features, this methodology is designed to unscramble hidden aspects in the data. As it was said, the algorithm will be used in two different tasks and applied in three different datasets: the density dataset contained both MLO and CC views for both left and right breast but, for each woman, only one view was available; the texture dataset contained cancer cases and controls, that were matched both for age and time of the first image available; finally, the Dutch breast screening dataset was composed of cancer cases and healthy controls and the same matching as before was made. As for the classification part, a two-layer neural network was used, with one layer being a previously used and trained convolutional layer, and the other being a SoftMax classifier, meaning that the previously learned parameters will be tuned through a supervised methodology. Broyden–Fletcher–Goldfarb–Shanno algorithm was used as optimizer, and 5-fold cross-validation was performed for a classification task that considers: “pectorales muscle and background”, “fatty tissue”, and “dense tissue” as labels for the density scoring; in what concerns texture scoring, “cancer” and “normal” are the considered labels. Regarding results, for density scoring, the output is a score, from 0 to 1, that represents the probability of a given pixel to belong to the “dense class”. Classification was done by choosing a value of probability to be a threshold, and the best results were obtained with a threshold of 0.75. The results explored the correlation between mammographic % PD done by the authors and by the radiologist, and a performance measure called Dice, that is given by:

(4) $$D=\frac{\left|A\cap B\right|}{\left|A\right|+\left|B\right|}$$

where *A* is the automated segmented region and B is the segmentation done by the radiologist. For this dataset, the correlation coefficient had a value of 0.85 (95% CI: 0.83–0.88), the Dice scores for fat and dense tissue were, respectively, of 0.95 ± 0.05 and 0.63 ± 0.19. The algorithm trained for this dataset was used to estimate % PD in the Dutch dataset and the cases had a value of 0.19 ± 0.11, the controls had a slightly smaller value—0.15 ± 0.11. The correlation of % PD between both breasts was of 0.93 (95% CI: 0.92–0.95) and the obtained AUC for differentiating cases and controls was of 0.59 (95% CI: 0.57–0.62). On the other hand, the texture scoring represents the probability of a given pixel to be a part of the cancer class. In order to get one texture score per image, the scores from 500 patches randomly selected across the breast area were averaged. Besides the developed algorithm, two other methods were used and evaluated in the performance of this task; one that is based in multiscale local jet features, and other that uses static histograms. To avoid bias, the algorithm was tested multiple times and outperformed the two well established mammographic texture scores, with an AUC of 0.61 (95% CI: 0.57–0.66) vs. AUCs of 0.60 and 0.56 (95% CI: 0.51–0.61) for local jet and static histogram approaches, respectively. Applying the algorithm to the Dutch dataset resulted in an AUC of 0.57 (95% CI: 0.54–0.61) in the differentiation between cases and controls and produced a correlation for the mammographic texture of 0.91 (95% CI: 0.90–0.92), between left and right breast. Based on their results for correlation and % PD based classification, the authors advoke that this methodology is close to the ones that are present in the scientific community and, based in the outperformance of their methodology in the texture scoring task, they proceed to state that this could be a better alternative to the handcrafted texture extraction that is the current state-of-the-art. One of the downsides of this approach is that the authors assumed that changes in mammography due to cancer occur in a generalized way across the breast, but the opposite can also be true, with texture changes being visible only in restricted areas, and an algorithm that could take this hypothesis into account should be considered as a future development of the considered work.

In 2014, Petersen et al. [44] sought to do breast segmentation and risk scoring using deep learning methodologies. Patients were considered as cancer cases for this study if they had had a screen-detected or an interval cancer; in the first case, mammograms four years prior from the diagnosis were considered; for interval cancer cases, mammograms from 2–4 years before cancer appearance were examined. Patient cases, as happens in other research, were age-matched with controls. An experienced radiologist rated the mammograms in BI-RADS scale and computed mammographic PD%. The model used, which is once again a convolutional sparse autoencoder, will learn features in an increasing abstraction fashion, in order to associate the computed set of features to the considered labels. The tasks that are going to be considered are: segmentation, with labels being “background”, “pectoral muscle”, “breast tissue”; % PD scoring, with the labels being “fatty tissue” and “dense tissue”; and texture scoring, with labels being “diseased” and “healthy”. As mentioned before, this methodology considers patches from different scales retrieved from the original image. The methodology for testing is the same as explained in the previous paper, but a special reference must be made to the fact that when the sliding window reaches the image border, the images is padded with constant values. The architecture used in this study is the same as used by Kallenberg, and the authors make a reference that, usually, convolutional and pooling layers are displayed in an alternate fashion, but in both studies, one of the pooling layers is replaced by another convolutional layer in order to grant the conditions of noise invariance and small-scale details sensibility. As for results, for segmentation, a dice metric was computed for an automated segmentation and a segmentation done by an expert and the results are: 0.99 ± 0.01, for background; 0.95 ± 0.08, for pectorales muscle; and 0.98 ± 0.01 to breast tissue. The correlation between automated and manual mammographic % PD scores was of 0.87, and the AUC for differentiating cases and controls, using the autoencoder was of 0.56 (95% CI: 0.51–0.61). For texture analysis, the performance of the algorithm for both left and right view were compared to a state-of-the-art texture scoring method and outperformed it (AUC = 0.65 [95% CI: 0.60–0.70] vs. 0.62 [95% CI: 0.57–0.67]). This research, although widely similar to the last one, proves that the used methodology generalizes relatively well to other datasets.

Back to 2016, when Qiu et al. [45] tried to create an algorithm that could, in an unsupervised fashion, estimate bilateral mammographic tissue density asymmetry, an important risk factor for the development of breast cancer. The authors aim to verify if this deep learning approach provides better results than the conventional machine learning methodology. For this study, each case had a “prior” and a “current” evaluation, all the prior mammograms were negative and the division between cancer and control cases was done based on the “current evaluation”. The algorithm developed aims to predict, based in the “prior” exam, the likelihood of a case (women) to have an image-detectable cancer in the “current” mammogram. The deep learning network proposed by the authors has 8 layers and can be divided in two subsets: feature learning set, and classification set. The first is composed by alternate convolutional and pooling layers, actually creating three convolutional-pooling pairs. Convolutional layers apply convolutional kernels to the input and, then, the pooling layers are responsible for granting that the bilateral asymmetry is size and rotation independent. After passing through the first pair, a 20 × 48 × 48 feature map is created, and, when passing through the following two pairs, the final result will be a 5 × 6 × 6 feature map, that is directly linked to the classifier—multiple layer perceptron—that will generate the probability of having an image detectable cancer in the next mammogram. During the training, with the 200 cases, a method called mini-batch statistic gradient descend was used to optimize the algorithm, which the authors say that provides better optimized parameters with lower computational effort. The algorithm was tested with the test set and evaluated through a confusion matrix and ROC curve analysis. This metrics allowed the authors to state that the specificity of the classifier was of 0.60, and the sensitivity achieved a value of 0.703. The AUC value was of 0.697 ± 0.063 and the overall accuracy, based on the confusion matrix, was of 71.4%. This methodology allowed to overcome the problem of manually choosing features to describe bilateral asymmetry once the features are optimal and directly learned from the input. The authors proceed to state that, even though the metrics to evaluate the algorithm provide confidence, this is yet an early study, with a small dataset that does not incorporate inter-women variations and that, having only 8 layers, is not deep enough, which are limitation that need to be overcome in order for this type of approaches to be considered in clinical practice.

Table 1, Table 2 and Table 3 present a summary of the works assessed in this review. The first addresses questions concerning dataset description and host Institutions, while Table 2 is more related to the methodology used. As for the final table, results and main conclusions are addressed. In this table, for studies with more than one AUC result, only the highest value is considered.

## 4. Conclusions

Mainly, the reviewed articles, in terms of extracted features, had in common three major groups (intensity-based, GLCM and RL), and then present many feature-group variations, with spectral analysis being also vastly considered. In what comes to the feature extraction procedure, older papers used a manually single-ROI approach, while more recent ML studies opted to diversify the region analyzed. Some authors used several ROIs across the breast, others segmented the breast in different regions and extracted features from them, and yet, some research consider the entire breast for feature extraction. In papers that compared their approach with the classical single-ROI methodology, authors usually find that their procedure outperformed the use of a unique ROI. This may happen because, considering breast tissue heterogeneity, a single region does not account for this diversity, and therefore a tissue characterization that takes into account the entire breast (or more than one region) appears to be more robust. In terms of classifiers, the papers varied widely, from LDA to SVM, passing through decisions trees and logistic regression. More studies should be performed to assess if there is a classifier that is clearly superior to others. Nonetheless, the LDA approach proposed in the first analyzed paper achieved higher results than the other three classifiers, which would point that this classifier, for this type of tasks might outperformed the others. However, the work proposed by Tan in 2019, that used an LDA, was outperformed by a work conducted in 2013, that used an SVM. As it can be perceived, there is not a clear conclusion to be made in terms of what classifier is the best. Nevertheless, the results obtained by the reviewed papers allowed to conclude that texture analysis along with machine learning algorithms can be correctly employed in risk analysis, either by differentiating risk groups, or by giving a risk score to each patient. Besides understanding that this type of methodology can be used, the research also points out that procedures that consider the entire breast for feature extraction might provide more useful information. While many of these studies were conducted in Caucasian population, the study presented by Tan in 2019 allowed to understand that ML algorithms and texture analysis can also be used, with good outcomes, in Asian populations. The results of the deep learning approaches, although lower than the ones presented by the classical ML approach, appear to be very promising, especially because dismisses the laborious work of extracting handcrafted features, and allows the possibility of automatically finding predictors that better serve the purpose of the study.

Given the articles discussed in this paper, excluding the ones that use deep learning, two great future endeavors should be examined: first, considering the substantial differences in age and other risk factors between high-risk and low-risk groups, studies should start using larger matched-groups and consider other risk factors than age, in an approach analogous to what was done in 2019 [38], but with more dataset cases; secondly, most of the papers did the validation of their model through cross-validation, meaning that training and testing samples came from the same dataset, so, novel studies should try to validate their models in an independent dataset. Machine learning methodologies are widely used in this area, which is demonstrated by the given publications’ date range considered here but should be interesting for new studies in the field of breast cancer risk assessment to consider deep learning, as it happens to the last three papers that were analyzed. Machine learning approaches proved to be substantially good in differentiating risk groups, but what might be more valuable in terms of medical application is the generation of risk scores, as done by Tan in 2013. The restriction to a high-risk/low-risk classification seems very limitative and the focus in giving a risk score specific to each woman should be considered.

While the development of new methodologies in both machine and deep learning, that suppress the weaknesses discussed in this section, might result in better outcomes, authors should start looking for breast cancer risk assessment in the perspective of transforming these algorithms and methods into real clinical applications.

The extensive review performed here allowed to have a general idea of what has been done for breast cancer risk prediction using textural analysis, that is sometimes combined with important risk factors. Although there are some downsides that can be pointed out to research’s methodologies, they serve as a proof of concept that parenchymal texture patterns provide important information about breast cancer risk and should, once methodology’s flaws are overcome, be used in clinical practice, and have a positive effect in millions of women that are diagnosed with breast cancer each year, worldwide.

## Figures and Tables

**Table 1:** Studies Data Summary.

Study	Institution	Mammogram View	Group-Matched?	Full Dataset Size
Hou et al., 2000 [18]	University of Chicago	CC	Yes. Age-matched	158 women = 15 high/143 low risk
Li et al., 2004 [27]	University of Chicago	CC	Yes. Age-matched	90 women = 30 high/60 low risk
Li et al., 2005 [30]	University of Chicago	CC	Yes. Age-matched	172 women = 30 high/142 low risk
Tan et al., 2013 [31]	University of Pittsburgh	CC	No.	645 women = 283 high/362 low risk
Zheng et al., 2015 [34]	University of Pennsylvania	MLO	Yes. Age-matched	424 women = 106 high/318 low risk
Tan et al., 2016 [37]	University of Pittsburgh	CC and MLO	Yes. Age-matched	335 women = 159 high/175 low risk
Tan et al., 2019 [38]	Subang Jaya Medical Center	CC	Yes. Age, Ethnicity, BMI-matched	500 women = 250 high/250 low risk
Gandomkar et al., 2020 [39]	Fudan University Shanghai	CC	No.	1079 women = 85 high/993 low risk
Kallenberg et al., 2016 [41]	University of Copenhagen	CC and MLO	Yes. Age and Acquisition time	Density: 493 healthy Texture: 226 cancer and 442 controls Dutch: 384 cancer and 1182 controls
Petersen et al., 2014 [44]	University of Copenhagen	MLO	Yes. Age-matched	495 women = 245 cases/250 controls
Qiu et al., 2016 [45]	University of Oklahoma	CC	No.	270 women = 135 cases/135 controls

**Table 2:** Methods Summary.

Study	ROI Analyzed	Intensity-Based	GLCM	RL	Other Features	Classifier/Algorithm
Hou et al., 2000 [18]	256 × 256, manually placed behind the nipple.	x	-	-	NGTDM, Spectral	LDA.
Li et al., 2004 [27]	256 × 256, 128 × 128 and 64 × 64 in referred locations	x	-	-	NGTDM, Spectral	ROCA.
Li et al., 2005 [30]	256 × 256, manually placed behind the nipple	x	x	-	Fractal, Spectral, Edge	ROCA.
Tan et al., 2013 [31]	Entire breast considered—segmented into regions.	x	x	x	Cumulative Projection	SVM.
Zheng et al., 2015 [34]	Lattice-based approach. D = W = 63, 127 and 255.	x	x	x	LBP, Fractal, Edge	Logistic Reg.
Tan et al., 2016 [37]	Entire breast considered	x	x	x	Weber, Structural sim.	SVM.
Tan et al., 2019 [38]	Entire breast considered	x	x	x	Structural, Spectral	LDA.
Gandomkar et al., 2020 [39]	Two segmented areas using AutoDensity	x	x	-	Fractal	Decision Tree
Kallenberg et al., 2016 [41]	Patches with the smaller scale being 4.8 mm × 4.8 mm and the biggest 3.7 cm × 3.7 cm.	-	-	-	-	Sparse autoencoder.
Petersen et al., 2014 [44]	Patches.	-	-	-	-	Sparse autoencoder
Qiu et al., 2016 [45]	256 × 256, manually placed behind the nipple	-	-	-	-	Multiple Layer Perception.

**Table 3:** Results Summary.

Study	AUC Results	Main Conclusion
Hou et al., 2000 [18]	AUC = 0.91	Mammographic features were found to be associated with breast cancer risk. High-risk women tend to have dense breasts and the patterns present e mammograms tend to have low contrast and to be coarse.
Li et al., 2004 [27]	AUC = 0.93 (highest value)	Features extracted immediately behind the nipple tend to have the best performance. Concerning size, results were not statistically significant.
Li et al., 2005 [30]	AUC = 0.66 ± 0.05 − 0.86 ± 0.03 (only assessed individual features)	High-risk women tend to have dense breasts and their pattern tend to be coarser, to have a lower fractal dimension, to be lower in contrast and to have a small edge gradient measure.
Tan et al., 2013 [31]	AUC = 0.716 ± 0.020 (first and third subgroup) AUC = 0.725 ± 0.018 (all groups)	Risk calculation based on texture features of mammographic asymmetry through a SVM classifier has a good potential to predict the near-term risk of breast cancer in women.
Zheng et al., 2015 [34]	AUC = 0.85 ± 0.02 (highest value)	Lattice-based approach allows parenchyma characterization across the entire breast, meaning that the extracted features are provide better information than the ones extracted from classic approaches.
Tan et al., 2016 [37]	AUC = 0.730 ± 0.027 (highest value)	Proved a relationship between the risk scores generated by the proposed model and the near-term risk of having breast cancer.
Tan et al., 2019 [38]	AUC = 0.68 (95% CI: 0.64–0.73)	Breast texture analysis has a great potential as an independent risk factor. The study used an Asian population and confirmed previous studies performed in Caucasian women about the relationship between texture patterns and breast cancer risk.
Gandomkar et al., 2020 [39]	AUC = 0.884 (CI 0.838–0.913)	A model that combines texture information and epidemiological factors might lead to an increased discriminatory capacity of risk prediction.
Kallenberg et al., 2016 [41]	Density: AUC = 0.59 (95% CI: 0.57–0.62) Texture: AUC = 0.61 (95% CI: 0.57–0.66) and 0.57 (Dutch) (95% CI: 0.54–0.61)	Obtained breast density scores are positively related to manual density scores, and texture scores have a predictive value in what concerns to breast cancer.
Petersen et al., 2014 [44]	AUC = 0.65 (95% CI: 0.60–0.70)	PMD scores correlate positively to manual scores and mammographic texture are more related to future breast cancer risk than scores related to mammographic density.
Qiu et al., 2016 [45]	AUC = 0.697 ± 0.063	This study concluded that deep learning technologies may have the potential to develop new risk predicting methods, that help to achieve an early detection of breast cancer through negative mammograms.

## Data Availability

Not applicable.
